# Zinc Transporter 8 (ZnT8) Expression Is Reduced by Ischemic Insults: A Potential Therapeutic Target to Prevent Ischemic Retinopathy

**DOI:** 10.1371/journal.pone.0050360

**Published:** 2012-11-27

**Authors:** Michael DeNiro, Futwan A. Al-Mohanna

**Affiliations:** 1 Research Department, King Khaled Eye Specialist Hospital (Affiliate of the Wilmer Eye Institute of the Johns Hopkins Medicine), Riyadh, Saudi Arabia; 2 Department of Comparative Medicine, King Faisal Specialist Hospital & Research Centre, Riyadh, Saudi Arabia; 3 Department of Cell Biology, King Faisal Specialist Hospital & Research Centre, Riyadh, Saudi Arabia; 4 Department of Cell Biology & Molecular Physiology, Medical College, Al-Faisal University, Riyadh, Saudi Arabia; Biological Research Centre of the Hungarian Academy of Sciences, Hungary

## Abstract

The zinc (Zn^++^) transporter ZnT8 plays a crucial role in zinc homeostasis. It’s been reported that an acute decrease in ZnT8 levels impairs β cell function and Zn^++^ homeostasis, which contribute to the pathophysiology of diabetes mellitus (DM). Although ZnT8 expression has been detected in the retinal pigment epithelium (RPE), its expression profile in the retina has yet to be determined. Furthermore, the link between diabetes and ischemic retinopathy is well documented; nevertheless, the molecular mechanism(s) of such link has yet to be defined. Our aims were to; investigate the expression profile of ZnT8 in the retina; address the influence of ischemia on such expression; and evaluate the influence of YC-1; (3-(50-hydroxymethyl-20-furyl)-1-benzyl indazole), a hypoxia inducible factor-1 (HIF-1) inhibitor, on the status of ZnT8 expression. We used real-time RT-PCR, immunohistochemistry, and Western blot in the mouse model of oxygen-induced retinopathy (OIR) and Müller cells to evaluate the effects of ischemia/hypoxia and YC-1 on ZnT8 expression. Our data indicate that ZnT8 was strongly expressed in the outer nuclear layer (ONL), outer plexiform layer (OPL), ganglion cell layer (GCL), and nerve fiber layer (NFL), whereas the photoreceptor layer (PRL), inner nuclear layer (INL) and inner plexiform layer (IPL) showed moderate ZnT8 immunoreactivity. Furthermore, we demonstrate that retinal ischemic insult induces a significant downregulation of ZnT8 at the message and protein levels, YC-1 rescues the injured retina by restoring the ZnT8 to its basal homeostatic levels in the neovascular retinas. Our data indicate that ischemic retinopathy maybe mediated by aberrant Zn^++^ homeostasis caused by ZnT8 downregulation, whereas YC-1 plays a neuroprotective role against ischemic insult. Therefore, targeting ZnT8 provides a therapeutic strategy to combat neovascular eye diseases.

## Introduction

Zinc (Zn^++^) is the second most abundant transition metal in mammals [1]. Since Zn^++^ is utilized in a number of biological processes [2], its homeostasis is tightly controlled on both; the systemic and cellular levels via different mechanisms [3]. Zinc homeostasis is regulated by an array of zinc transporters, controlling its movement from the extracellular space to the cytosol, and from the cytosol to intravesicular space. Two types of transporters exist; zinc transporter (ZnT) and Zrt- and Irt-like proteins (ZIP). There are 10 ZnTs (*SLC30A*) and 14 ZIPs (*SLC39A*), which have been identified in mammals with different tissue expression, cellular localization and regulation [4], and their expression is dependent on tissue and cellular status. The SLC30A efflux is localized in the membrane of the insulin secretory vesicles and it facilitates the accumulation of zinc from the cytoplasm into the insulin-containing vesicles. It plays a major role in providing zinc for insulin maturation and/or storage processes [5]. Whereas the SCL39A influx transporter family, Zrt-Irt-like Protein (ZIP) acts in an opposing manner to increase intracellular Zn^++^ levels [6]. A major role of Zn^++^ in the pathogenesis of DM is not surprising since Zn in β cell secretory vesicles is essential for insulin hexamerization. This is supported by the findings that polymorphism in a genetic variant of ZnT8 (*SLC30A8*) is associated with increased risk of type 2 DM (T2DM) [7]. Furthermore, ZnT8 (*SLC30A8*) has been reported as an auto-antigen and a major susceptibility gene for type 1DM (T1DM) and T2DM, respectively. These autoantibodies are present in 60–80% of new cases of T1DM [8]. Dysregulation in Zn^++^ homeostasis is firmly implicated in the pathophysiology of many acute neural injuries and chronic neurodegenerative diseases. In addition, perturbations in Zn^++^ homeostasis disrupt carbohydrate metabolism; however, the inverse relationship is also true: DM and hyperglycemia alter Zn^++^ balance, promoting hypozincemia and hyperzincuria [9]. The importance of Zn^++^ in biological processes is crucial to housekeeping proteins, cellular metabolism, and gene expression. It provides structural stability to the Zn^++^ finger domains of many DNA-binding proteins and is a cofactor for more than 300 metalloenzymes, in which it is an essential element for the catalytic and/or structural integrity. Beta cells have insulin-containing vesicles, which also contain high intracellular concentrations of Zn^++^. Furthermore, Zn^++^ facilitates the packaging of insulin into hexamers through two Zn^++^ ions, a step necessary for insulin crystallization [10], [11], [12].

Chimienti and co-workers originally described the zinc transporter encoded by *SLC30A8*; ZnT8, as a pancreatic islet-expressed protein is associated with the ZnT family of intracellular Zn^++^ transports [13]. Loss of ZnT8 from pancreatic β cells reduces insulin content and compromises insulin release. Interest in the role of zinc in the pathogenesis of DM was re-erupted with the discovery of the association between T2DM and a genetic polymorphism in the *SLC30A8* gene [14], [15]. This polymorphism, which is caused by the minor allele of the single-nucleotide polymorphism rs1226634 (C/T transition; Arg {325}–Trp 273 {325}) [16], was subsequently shown to be associated with the presence of altered glucose homeostasis, pancreatic β cell dysfunction, or overt T2DM in many [17], [18] but not all [19], [20] study populations. Furthermore, the insulin producing pancreatic β cells contain some of the highest levels of Zn^++^ in the body. This high Zn^++^ content is largely due to the critical function of Zn^++^ for insulin synthesis, secretion and signaling [21], and the role that may play in the protection against oxidative stresses [22]. Zinc deficiency may predispose individuals to DM and its cardiovascular complications [23]. Overall, ZnT8 may contribute to the pathogenesis of DM due to autoantigenic properties as well as decreased protein function, which may be exacerbated by polymorphic variance.

Zn^++^ plays an essential role in the retinal function; this is echoed by its relatively high content in ocular tissue with the retinal being the highest [24]. It has been revealed that the RPE and the choroid contain the highest levels of zinc concentrations in the retina. Several reports addressed the role of Zn^++^ in oxidative damage to the retina [25]. In addition interactions between Zn^++^ and the antioxidant amino acid taurine have long been addressed [26]. Recent studies have indicated that the intracellular localization of Zn^++^ pools in photoreceptors changes with light exposure, with the greatest intensity of Zn^++^ staining observed in the perikarya of photoreceptors of dark-adapted retinas and in the inner segments of light-adapted retinas. Furthermore, the expression of ZnT8 has been detected in the primary human fetal RPE cultures and human ARPE19 cell line; whereas pigment epithelium-derived factor (PEDF) induced a strong increase of ZnT8 mRNA levels in these cells [27]. In addition, it has been reported that ZnT8 is upregulated in the RPE cell layer located in the mouse retina of the *Hfe*−*/*− mouse [28]. The *Hfe*−*/*− mouse is a knockout mouse model of hereditary hemochromatosis; a common autosomal recessive disease characterized by increased iron absorption and progressive iron storage that results in damage to major organs in the body. The *Hfe*−*/*− mouse exhibits profound differences in parameters of iron homeostasis.

Given the importance of tightly-regulated zinc homeostasis for normal retinal cell physiology, we examined whether the effects of ischemia/hypoxia on Müller cells, *in vivo* and *in vitro*, may be mediated, in part, through altered expression of zinc transporters. This study aims to address the impact of ischemic injury on the expression of ZNT-8 in glial cells and the ischemic retina. This investigation has also examined the effects of YC-1 treatment on the expression of ZnT8 transporter expression in the Müller cell line (rMC-1) and the injured ischemic retina.

## Materials and Methods

### Ethics Statement

All experiments were conducted in compliance with the laws and the regulations of the Kingdom of Saudi Arabia. In addition, all animal protocols were approved by the Institutional Review Board and conformed to the ARVO Statement for the Use of Animals in Ophthalmic and Vision Research statement of the Association for Research in Vision and Ophthalmology.” All surgery was performed while the animals were under ketamine and xylazine anesthesia, and all efforts were made to minimize suffering. This research study was approved by: “**THE KING KHALED EYE SPECIALIST HOSPITAL’S HUMAN ETHICS COMMITTEE & INSTITUTIONAL REVIEW BOARD (HEC/IRB), RIYADH, SAUDI ARABIA**”. The permit number/approval ID is “RP 0630-P”.

### Reagents

Anti-rat/anti-mouse ZNT-8 polyclonal antibody (RZ8) was purchased from Mellitech (Grenoble cedex- FRANCE). Polyclonal rabbit anti-β-actin antibody was purchased from MBL international (Woburn, MA). Goat anti-rat IgG was purchased from Bethyl Lab (Montgomery, TX) was used as an isotype control antibody for Western Blot studies. Rat anti-Mouse IgG was purchased from eBioscience (San Diego, CA) was utilized for immunohistochemistry studies. YC-1 was purchased from A.G. Scientific (San Diego, CA) and dissolved in sterile DMSO.

### Tissue Culture

The rat Müller cell line (rMC-1) [29] was kindly provided by Dr. Vijay Sarthy (Northwestern University, Evanston, IL, USA). Müller cell cultures were grown in DMEM supplemented with 15% FBS, and a fungicide mixture and 0.5% gentamicin, incubated in a humidified atmosphere of 5% CO_2_/95% air. Medium was changed every 2 days, and cells were grown to confluence in a 150-mm dish. Cells were split into 60-mm dishes and were used in the experiments when confluent.

### *In Vitro* Hypoxia

Inducing hypoxia *in *vitro was conducted as previously described [30]. Briefly, cells were placed in airtight chambers (BioSpherix, Redfield, NY) and the O_2_ tension was maintained at 1.2% by using Pro-Ox Model 110 O_2_ regulator (BioSpherix, Redfield, NY). The chamber was purged with a gas mixture of 5.32% CO_2_, and 93.48% N_2_.

### Quantitative Real-Time RT-PCR

**Figure 1:**
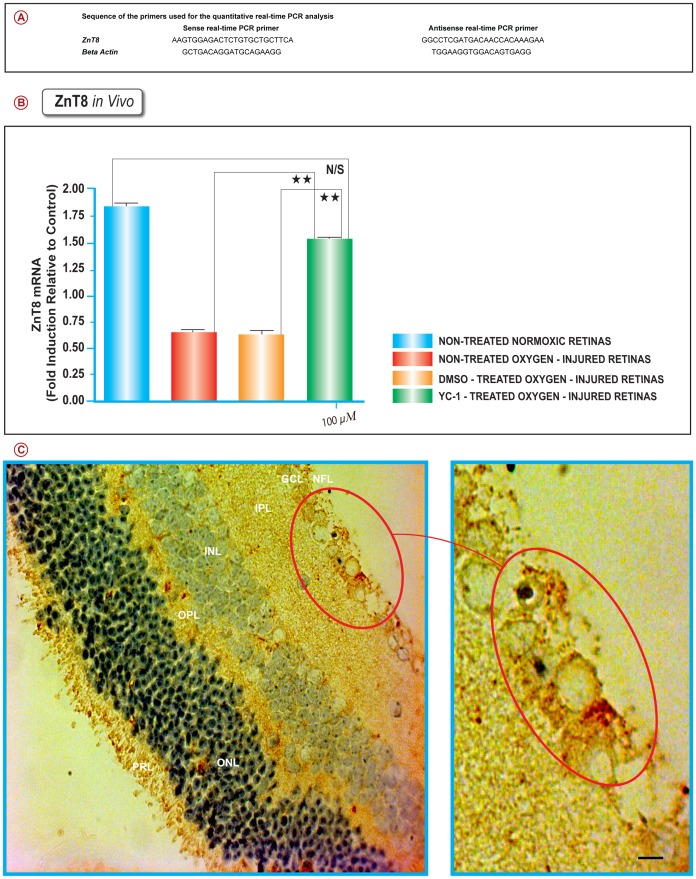
YC-1 Curtails Ischemia-Induced Downregulation of Basal ZnT8 Expression in the Injured Retina. Quantitative Real-Rime RT-PCR analysis was conducted by utilizing specific primer sets (A). The levels of *ZnT8* mRNA in the non-treated ischemic retinas were significantly downregulated by approximately 3.05 folds, as compared to nontreated normoxic retinas. Dual injections treatment with YC-1 has resulted in a significant upregulation of *ZnT8* (**P<0.01) gene expression when compared with DMSO-treated hypoxic cells. ANOVA; Mean ± SEM of mRNA level normalized to β-actin were calculated, (**P<0.01, as compared to DMSO-treated retinas). Data are representative of 3 independent experiments (B). Immunohistochemical localization of ZnT8 in the Mouse Retina has indicated that in the non-treated normoxic retina; the ONL, OPL, GCL, and NFL tissue layers of the retina exhibited the strongest ZnT8 expression, whereas the PRL, INL and IPL exhibited moderate ZnT8 immunoreactivity. An image with high level of magnification (marked with circle) shows staining of the cell bodies in the GCL. Scale bar: 200 µm (C).

Total cellular RNA was isolated using TriZol reagent (Invitrogen). Total RNA (5 µg) was used to generate cDNA with the SuperScript III First-Strand Synthesis System (Invitrogen); product (3 µl) was amplified with TaqMan Universal PCR Master Mix (Applied Biosystems) on a StepOne Plus platform (Applied Biosystems). We used the primers summarized in (Fig. 1A) for RT-PCR. Quantitative Real-Rime RT-PCR was conducted, as previously described [30]. Briefly, gene-specific molecular beacons and primers were designed to encompass the genes of interest, with beacon’s annealing site to overlap with the exon-exon junctions for additional specificity (Beacon Designer 6.0, Premier Biosoft International, Palo Alto, CA, USA). Threshold cycle (Ct) values for the different samples were utilized for the calculation of gene expression fold change using the formula 2 to the minus power of delta delta ct. Fold changes in the *ZnT8* gene relative to the *β-actin* endogenous control gene were determined by the following equation: fold change  = 2^–Δ (Δ*C*^_T_^)^, where change in threshold cycle (Δ*C*_T_) = *C*_T_ (*gene of interest*) – *C*_T_ (*β-actin*) and Δ (Δ*C*_T_) = Δ*C*_T_ (treated) – Δ*C*_T_ (untreated).

### Western Blot

Cells were seeded overnight in 6-well plates (10^5^ cells/well). Müller cells (rMC-1) were treated with either YC-1 (25–100 µM) or DMSO (0.2% *v/v*) for 48 hr under normoxic or hypoxic environments. Reactions were terminated by addition of lysis buffer (Cell Signaling, Beverly, MA). Western Blot analysis was conducted, as previously described [31]. Briefly, protein content of the cell lysates was determined according to the Bradford method (Bio-Rad, Hercules, CA). Aliquots (40 µg) of whole-cell lysates were separated on 7.5% SDS-PAGE, and electro-transferred onto polyvinylidene membranes (Amersham Pharmacia Biotech, Little Chalfont). After blocking with 5% nonfat dry milk in TBS-T, the blots were incubated overnight with anti-(ZnT8, and β-actin {internal control}) antibodies. Negative control experiments consisted of omission of the ZnT8 antibody and utilizing a goat anti-rat IgG (isotype control antibody) as a replacement. Then blots were washed 3 × 10 min washes in PBS/tween and subsequently incubated with peroxidase-conjugated anti-mouse IgG secondary antibody at 1∶3000. The signals were obtained by enhanced chemiluminescence (Amersham Biosciences), and visualized by exposure to X-ray film. Upon completion of chemiluminescence, equal lane loading was checked by Ponceau S Solution (Sigma, St. Louis, MO). X-ray films were scanned with a computer-assisted densitometer (model G-710; Bio-Rad) to quantify band optical density (Quantity One software; Bio-Rad).

### Care and Treatment of Mice

OIR was induced in litters of C57/BL6J mice (The Jackson Laboratory), as previously described [32]. Retinal neovascularization (NV) was induced in newborn mice as described previously. Briefly, P7 mice were exposed with their nursing mother, for 5 days (between P7 and P12) to hyperoxic conditions, by incubating them in an airtight chamber (PROOX 110 chamber O_2_ controller; Biospherix Ltd., Redfield, NY) ventilated by a mixture of O_2_ and air to a final oxygen fraction of 75±2%. These incubation conditions induced vaso-obliteration and subsequent cessation of vascular development in the capillary beds of the central retina. At P12, the mice were allowed to recover in normal room air conditions and maintained for another 5 days (till P17), the day in which peak disease occurs. A condition of relative hypoxia resulted between P12 and P17, and extensive retinal NV developed in 100% of the mice. Age-matched animals with the hyperoxia-exposed groups were maintained identically, except they were exposed to room air (21% O2, 79% N2) for the duration of the experiment. Animals were examined and sacrificed on the same days.

Mice were treated in accordance with the recommendations of the Association for Research in Vision and Ophthalmology (ARVO) and all animal protocols were approved by the Institutional Review Board (IRB). Mice were divided into four groups; (1) Non-treated mice grown under normoxic conditions; (2) non-treated hyperoxia-exposed mice; (3) DMSO- treated hyperoxia-exposed mice; and (4) YC-1-treated hyperoxia-exposed mice.

### Intravitreal Drug Injections

A group of hyperoxia-exposed animals (n = 15) were injected intravitreally (into both eyes) at P12 and P15 with 3 µl of YC-1 (100 µM) (drug-treated group). Another group of hyperoxia-exposed mice (n = 15) were injected intravitreally (into both eyes) at P12 and P15 with 3 µl of DMSO (0.2% {v/v}). Non-treated mice grown under ambient conditions, non-treated hyperoxia-exposed mice, DMSO- treated hyperoxia-exposed mice and YC-1-treated hyperoxia-exposed mice, were all examined at different critical time points for qualitative assessment of the retinal vasculature by fluorescein angiography.

### Immunohistochemistry

Mouse retinas were dissected and prepared for immunohistochemical analysis, fixed in 4% paraformaldehyde in 0.1 M PBS for 15 min at room temperature and embedded in paraffin, sectioned (5 µm). Tissue sections were deparaffinized, hydrated, and later exposed to heat-induced antigen retrieval using a microwave oven (three 5-min cycles in citrate buffer, pH 6.0), endogenous peroxidase was abolished with methanol, and hydrogen peroxide and nonspecific background staining was blocked by incubating the tissue sections for 5 min in normal swine serum. Subsequently, all slides were washed three times in PBS, and incubated for 1 hr with primary anti–(ZnT-8 and β-actin) antibodies. Negative control experiments consisted of omission of the ZnT8 antibody and utilizing a rat anti-mouse IgG (isotype control antibody) as a replacement. The sections were washed with TBST and incubated with EnVision Polymer HRP secondary antibody (DAKO, Carpinteria, CA) for 30 min. All slides were stained with DAB solution and counterstained with hematoxylin. Slides were cover slipped (Permount; Fisher Scientific, Fairlawn, NJ) and examined by light microscopy. Negative controls included omission of the primary antibody or its substitution with phosphate-buffered saline (PBS). Sections were photographed under a microscope (Zeiss Axiovert 135, Thornwood, NY), and images were acquired a digital camera (AxioCam, NY). All retinas were examined at X60 objective. The staining intensity in our series ranged from a weak blush to moderate or strong. The amount of cells staining with the antibody was further categorized as focal (<10%), patchy (10%–50%), and diffuse/multifocal (>50%). For meaningful semiquantitative analysis, focal and/or weak staining was considered equivocal staining, and patchy or diffuse/multifocal staining was either subcategorized as either moderate or strong staining. All immunohistochemical analyses were measured by Metamorph digital image software (Molecular Devices, Sunnyvale, CA).

### Immunohistochemical Image Analysis

ZnT8 positive immunostaining were captured using AxioCam digital microscope camera. MetaMorph image analysis was conducted by setting the filter with excitation wavelengths 488. MetaMorph image analysis software (version 7.1, Universal Imaging, Downingtown, PA) was used for image processing and quantitative analysis of ZnT8 positive immunostaining. MetaMorph tools were used to set the threshold and regions of interest (ROIs). All images were captured at identical time and exposure settings, and they were all processed to the same scale. Images were first segmented on the basis of pixel intensity, which was done on a plane-by-plane basis for an image stack. Briefly, each retinal section was scanned into Metamorph and five (5) fields/slide was chosen from each section for analysis. One hundred and fifty (150) cells from each field were selected. The saved file was used to calibrate each image for specific pixel size. With the help of a free drawing tool, ZnT8-stained areas were chosen and measured in total-pixels area. A threshold encompassing an intensity range of 100–250 gray-scale values was applied to the ROIs in the least brightly stained condition first. The data were also read and investigated by Matlab v6.5 script file software, which counted the total number of pixels that were above threshold value. This number was divided by the total number of pixels in each image to yield percent fluorescent pixels. To correct for background fluorescence, the threshold was adjusted for each experimental series, with concomitantly processed negative controls used as the guide for setting background fluorescence. The background fluorescence intensities per pixel were subtracted from the experimental data by using a one-step erosion procedure, and then all remaining objects were counted. The same threshold was subsequently applied to all images. ZnT8 was considered to be positive only when it exceeded the established threshold. Percent ZnT8 expression above threshold in the total area selected was then calculated. The total ZnT8 fluorescence intensity per cell was calculated, and the average fluorescence intensity per pixel was determined by dividing the total intensity by the area of the cell measured in pixels. This was followed by measuring the average fluorescence intensity in each field. Data from multiple fields as indicated over several experiments were used to obtain the final results. The number of immunopositive-stained cells per image was then expressed per um^2^, and the average number per section was determined among five separate fields.

### Statistical Analysis

Analysis was performed utilizing ANOVA for multiple variables and with *t–*Tests for comparison of 2 groups with normal distribution. For the analysis of Real Time RT-PCR data; immunohistochemistry data; Western Blot data, analysis was performed with ANOVA for multiple variables and with *t–*Tests. Data are expressed as mean ± SEM from at least 3 independent experiments. Significance was defined as *P<0.05; **P<0.01; ***P<0.001.

## Results

### Ischemic Insult Mediates a Significant Downregulation of ZnT8, *in vivo*

The retinal ZnT8 mRNA gene expression levels were evaluated on P17, by real time RT-PCR. Data were normalized to β-actin mRNA levels. By using the primers shown in Fig. 1A; our data revealed that there was a significant (**P<0.01) downregulation in the message levels in the non-treated oxygen-injured retinas (Fig. 1B: Red Bar) (2.96±0.02) and DMSO-treated retinas (Fig. 1B: Orange Bar) (3.08±0.01) as compared with the retinas from animals that were placed under ambient conditions (Fig. 1B: Blue Bar) and retinas from animals that were treated with 100 µM YC-1 (Fig. 1B: Green Bar). Therefore, the effects of sham treatment on the ZnT8 gene expression patterns paralleled those seen in the ischemic group.

### Characterization of ZnT8-immunoreactivity in the Mouse Retina

**Figure 2:**
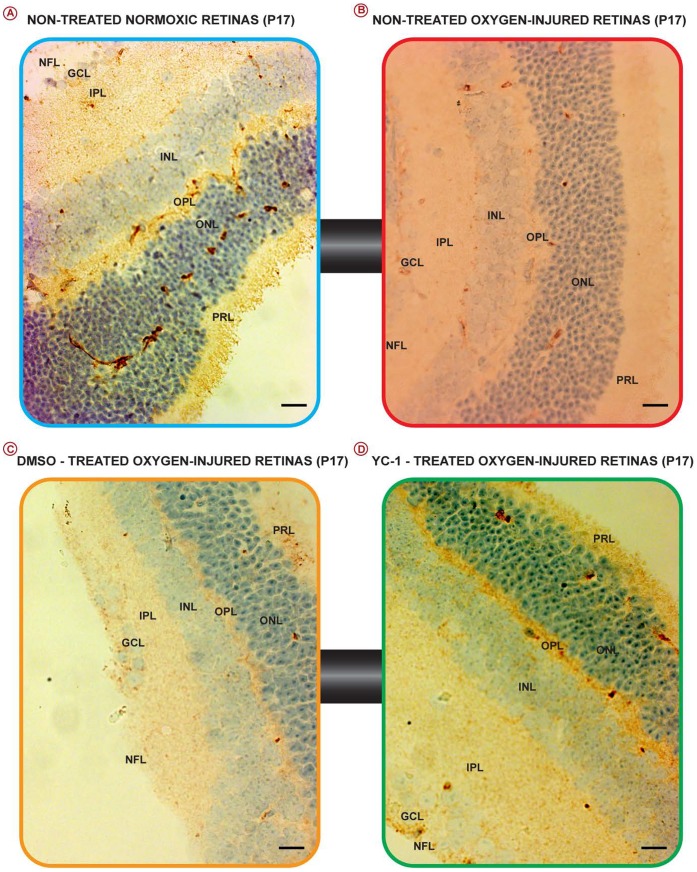
Characterization of ZnT8-immunoreactivity in Normal, Pathological and YC-1-Treated Retinas. Photomicrographs of retinas from various OIR groups that were immunostained for ZnT8 exhibit a downregulation of ZnT8 expression in the non-treated ischemic (B) and DMSO-treated groups (C), as compared with non-treated normoxic group (A). While ZnT8 immunoreactivities were upregulated in the YC-1-treated group (D), as compared with DMSO-treated groups. Data are representative of 3 independent experiments. Scale bar: 150 µm.

**Figure 3:**
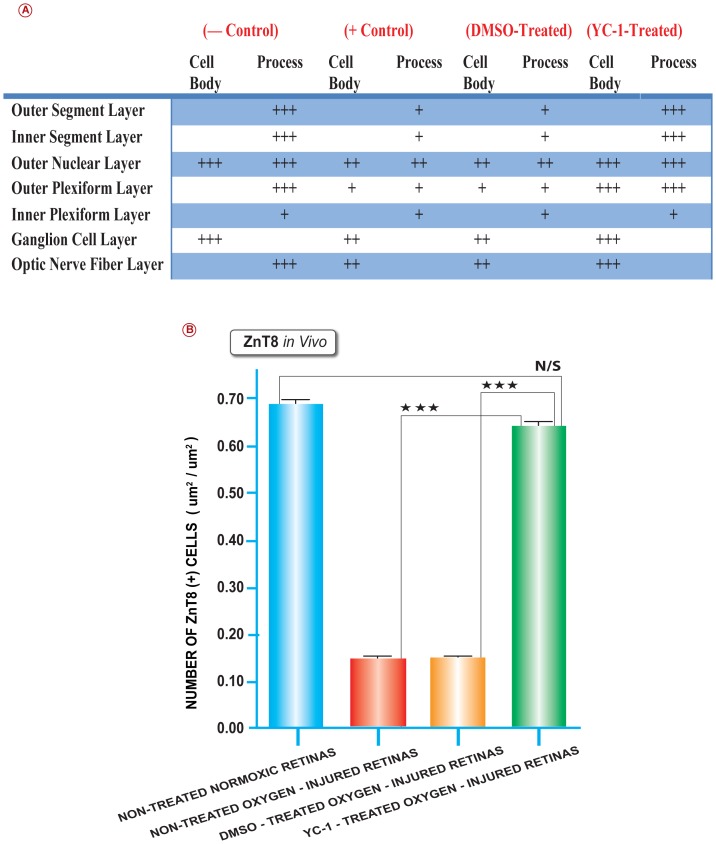
Quantitative Assessments of Retinal Immunohistochemical Staining Intensity of ZnT8 in Normal, Pathological and YC-1-Treated Retinas. The retinal layers stained vividly. However, the grain intensity varied significantly from one layer to another. The intensity of immunoreactivity was graded as follows: strong (+++), moderate (++), weak (+), negative (−) (A). Retinal tissue specimens of YC-1 treated groups were compared to normoxic, non-treated ischemic and DMSO-treated retinas. The collected images of the retinas were imported to the image analysis system Metamorph 7.1. All image analyses were conducted in a masked fashion. Values obtained from at least 5 retinal fields were used to calculate the average pixel intensity value per retina. Bar graphs exhibit the intensity of staining of ZnT8 in all four groups. The area of staining was measured in (um^2^/um^2^) in all four groups. Values (mean ± SEM), from 3 separate experiments from at least 10 images from 4 different eyes/group. (***P<0.001 and **P<0.01). Data are representative of 3 independent experiments (B).

Our immunohistochemistry data have indicated that the ONL, OPL, GCL, and NFL tissue layers of the normoxic retinas exhibited the strongest ZnT8 immunoexpression, whereas the PRL, INL and IPL showed moderate ZnT8 staining (Fig.1C; 2A; 3A; 3B). Based on their location, these cells were presumed to be amacrine and horizontal cells. Furthermore, since both NFL and INL exhibited ZnT8 reactivity; this suggests that both the axons and dendrites of ganglion cells contain ZnT8. Our immunohistochemical data have also demonstrated that ischemic insult has mediated a significant downregulation of ZnT8 at the protein level, *in vivo* (Fig. 2B; 3A; and 3B). The ZnT8-depleted cells were primarily localized in the photoreceptor layers/neurosensory retina (Fig. 2B; 2C). The nontreated O_2_-injured (Fig. 2B) and the DMSO-treated O_2_-injured retinas (Fig. 2C) exhibited a weak “focal”, sporadic staining signals for ZnT8 reactivity, which was primarily localized in the PRL, ONL, INL, GCL and the NFL regions. Ischemic injury has inflicted a significant (**P<0.01) downregulation of ZnT8 protein levels in the non-treated ischemic (5.38±20) as compared to normoxic retinas. In addition, ZnT8 expression was significantly (**P<0.01) downregulated in the DMSO-treated retinas (5.22±0.01) as compared with the retinas from animals that were placed under ambient conditions (Fig. 3B).

### YC-1 Curtails Ischemia-Induced Downregulation of Basal ZnT8 Expression at the Message and the Protein Levels, *in vivo*

Our data have demonstrated that treatment with a double intravitreal injection-regimen of YC-1 (100 µM) on P12 and P15 resulted in significant (**P<0.01) upregulation in the ZnT8 message level as compared with DMSO-treated ischemic retinas, and its expression level was comparable to that of the normoxic group (Fig. 1B: Green Bar). Furthermore, YC-1 treatment restores the ZnT8 expression to basal homeostatic level, which was comparable to those of the nontreated normoxic retinas (Fig. 2D; 3A; 3B). YC-1-treated retinas displayed a significant upregulation in ZnT8 immunoexpression as compared to DMSO-treated retinas. YC-1-treated retinas exhibited a significant (**P<0.01) elevation of ZnT8 expression and strong staining signals of ZnT8 expression, primarily in the ONL, OPL, GCL and NFL, whereas the PRL, INL, IPL expressed moderate levels of ZnT8 immunoreactivity. The staining intensity of ZnT8 in the YC-1-treated retinas was strong and significantly elevated by 5 folds as compared with DMSO-treated-injured retinas.

### Hypoxia Induces ZnT8 Depletion in Müller Cells, Whereas YC-1 Restores Basal ZnT8 Homeostasis, *in vitro*

**Figure 4:**
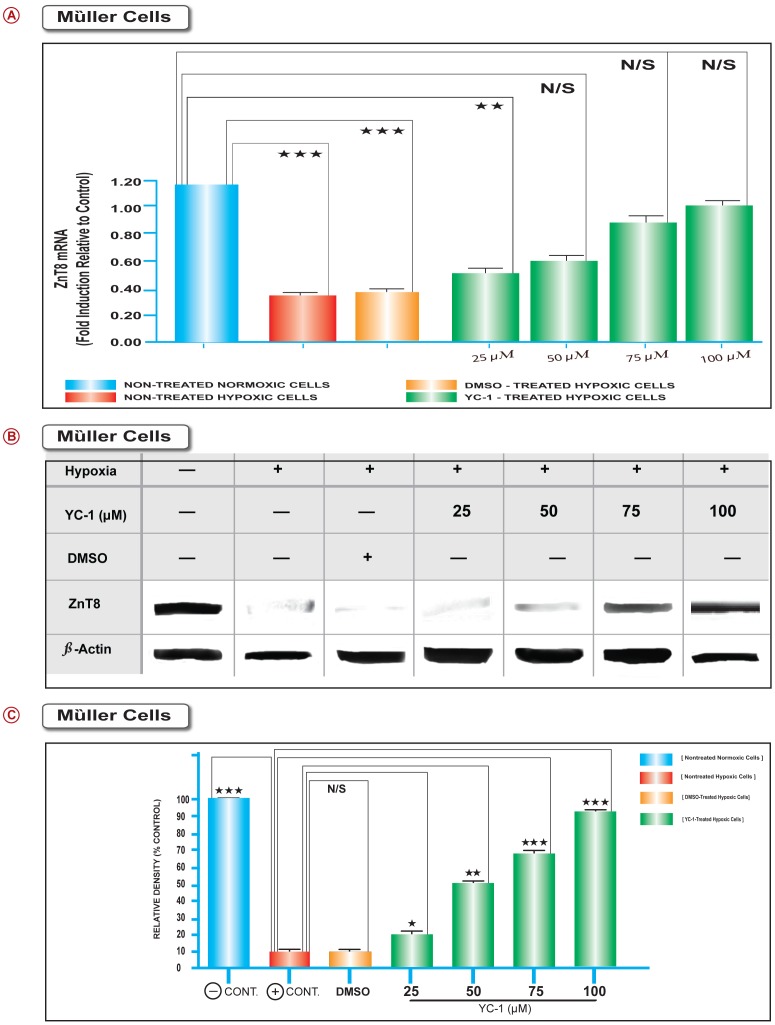
YC-1 Restores ZnT8 to its Basal Homeostatic Levels, *in vitro.* rMC-1 cells were cultured under normoxic and hypoxic conditions. The mRNA levels of *ZnT8* were downregulated in non-treated hypoxic cells; while normoxic cells exhibited remarkable high mRNA levels. Treatment of hypoxic rMC-1 cells with various concentrations of YC-1 resulted in a significant upregulation of *ZnT8* mRNA expression as compared to DMSO-treated hypoxic cells. ANOVA was used for statistical analyses. Mean ± SEM of mRNA level normalized to β-actin were calculated, (***P<0.001 and **P<0.01 as compared to DMSO-treated hypoxic control). Data are representative of 3 independent experiments (A). The protein expression levels of ZnT8 were significantly elevated in the normoxic cells, as compared to cells that were exposed to hypoxic conditions. In YC-1-treated hypoxic cells, ZnT8 protein expression was significantly increased in a dose-dependent fashion, compared with DMSO-treated hypoxic cells, which exhibited a significant ZnT8 downregulation. Statistical significance was determined by ANOVA (**P<0.01). Data are representative of 3 independent experiments (B). Graphs exhibit the densitometric analysis relative to that measured in non-treated hypoxic control. Relative ratio represented the intensities of ZnT8 protein expressions in rMC-1 cells relative to those of β-actin expression, whereas the relative ratio of normoxia control was defined as 100. Values, shown as the mean ± SEM, from 3 separate experiments with a total sample size of 6. (*P<0.05 **P<0.01 ***P<0.001) vs. DMSO-treated hypoxic control (C).

Since the above data have revealed that ZnT8 was primarily expressed in the PRL, ONL, OPL, GCL, and NFL tissue layers of the normoxic and YC-1-treated retinas. Ergo in view of the fact that glial cells extend from the INL to the outer limiting membrane (OLM); the possibility existed that YC-1 maybe acting directly on retinal glial cells. We therefore, used rMC-1 cells (Müller cell line) to study the direct effects of YC-1 on *ZnT8* mRNA expression. Real time RT PCR was employed to investigate the changes of *ZnT8* mRNA expression in the Müller cells. Post-exposure to hypoxia for 48 hrs, rMC-1 cells (Fig. 4A: Red Bar) revealed statistically significant downregulation in *ZnT8* mRNA level as compared to cells cultured under normoxia, which displayed high *ZnT8* mRNA levels (Fig. 4A: Blue Bar). Under hypoxic conditions, the expression of *ZnT8* was decreased by 3.1±0.7 folds, compared to normoxia. Treatment with DMSO did not influence the levels of *ZnT8* expression (Fig. 4A: Orange Bar). The expression of *ZnT8* in DMSO-treated rMC1 cells was 3.02±0.2 folds lower than normoxic controls (Fig. 4A: Orange Bar). Treatment of rMC1 with 25, 50, 75, and 100 µM YC-1 resulted in a significant dose-dependent upregulation of *ZnT8* expression under hypoxia, as compared with the corresponding DMSO-treated hypoxic control cells (Fig. 4A: Green Bar). Data were normalized to β-actin mRNA levels. As shown in Fig. 4A, real-time PCR assay elucidated the expression of *ZnT8* was significantly suppressed by hypoxic conditions, whereas the inhibitory effect was dramatically compromised and reversed in the presence of the HIF-1 inhibitor, YC-1. Taken together, our Real time RT-PCR data have revealed that post hypoxic exposure, the level of *ZnT8* mRNA expression was significantly (***P<0.001) downregulated, as compared to the normoxic control (Fig. 4A: Red Bar). DMSO-treatment had no effect on the *ZnT8* gene expression pattern in rMC-1 cells and it paralleled those in nontreated hypoxic cells (Fig. 4A: Orange Bar).

Western immunoblot analysis was performed. rMC-1 cells cultured under normoxia exhibited high signals of ZnT8, while this signal was downregulated after 48 hrs of hypoxic exposure as compared to normoxic control (Fig. 4B). In the DMSO-treated hypoxic cells, ZnT8 protein expression profile was compatible to the ZnT8 expression that was characterized in non-treated hypoxic cells. YC-1 treatment induced an augmentation in the hypoxia-downregulated ZnT8 protein levels in a concentration-dependent fashion, as compared to DMSO-treated hypoxic cells (Fig. 4B and 4C). In the DMSO-treated cells, ZnT8 protein levels remained comparable to non-treated cells that were cultured under hypoxic conditions. Since YC-1 treatment did not inhibit β-actin, this indicates that YC-1 influence on ZnT8 protein expression was specific. Our densitometry analysis indicated that ZnT8 protein expression was significantly downregulated in hypoxic cells, as compared to cells incubated under normoxic conditions (Fig. 4C). Direct measurements of protein levels by Western blot indicated that under hypoxic conditions; ZnT8 protein expression was significantly decreased by 10 fold, compared to normoxia; whereas treatment with 100 µM significantly upregulated ZnT8 expression by 8 folds in rMC-1 cells, compared to non-treated hypoxic cells, (***P<0.001) (Fig. 4C).

## Discussion

It has been determined that Zn^++^ dyshomeostasis, both systemically and in the pancreas, plays an intricate role in the pathology of both, T1DM) and T2DM [33]. Severe Zn^++^ deficiency induces hyperglycemia and hyperinsulinemia [34], directly implicating Zn^++^ in systemic glucose regulation. Consistent with a role for Zn^++^ in glycemic dysregulation, Zinc supplementation ameliorates some physiological symptoms of DM [34].

Diabetic retinopathy (DR) is one of the major causes of blindness world-wide. In this study, we have employed the OIR mouse model for various reasons: 1) This model depicts the human retinopathy of prematurity (ROP); 2) It represents an ischemia-dependent model, which manifests the overexpression of HIF-1, the main causation of retinal ischemia; 3) It manifests retinal vasculopathy, i.e., the development of retinal NV; 4) Ischemia has been implicated in the pathophysiology of age related macular degeneration (AMD) [35]. Recent findings strongly suggest that ischemia and vascular impairment play a central role in the etiology of AMD [36]; 5) Most importantly, zinc deficiency is highly associated with retinal pathologies that are induced by retinal ischemia. There is hoarding evidence, which indicate that zinc deficiency in the retina contribute to the pathogenesis of AMD. It has been suggested that replenishing zinc by oral administration had a positive effect on AMD patients by slowing down the progress of the dry form of the disease [37]. These findings support the view that zinc deficiency is involved in the pathogenesis of AMD. The 2004 AREDS report and other studies confirm that replacing zinc with a dietary supplement has beneficial effects against AMD [38]. Therefore, the Zn-AMD connection suggests that the risk for or severity of AMD increases with the depletion of available intracellular zinc pools in the retina [38].Hypoxia inducible factor members have been implicated in regulating the angiogenic and metabolic response to ischemia. HIF-1α is known to exist in subretinal NV and hypoxia is the main inducer for the production of vascular endothelial growth factor (VEGF) [39]. It has been reported that zinc hampers hypoxia-stimulated HIF-1 activation in astrocytes by inhibiting nuclear HIF-1α translocation and subsequently disrupting HIF-1 heterodimerization [40]. Zinc inhibited HIF-1α recruitment onto VEGF promoter and the zinc-induced suppression of HIF-1-dependent activation of VEGF. Of note is the finding that zinc also inhibited HIF-2α other than HIF-1α; this is highly relevant in light of the fact that recent studies have shown that HIF-2α may be the main regulator of long term (chronic) hypoxic gene expression [41].

In order to investigate the molecular and cellular mechanisms that influence the expression of ZnT8 under ischemic conditions; we have utilized the mouse model of OIR. This model has been widely used in studies related to proliferative DR, ROP, and in studies evaluating the efficacy of antiangiogenic compounds. In the retina, hypoxia may occur as a result of vascular disruption caused by various pathologies, such as hyperglycemia in DM, thrombosis in vein occlusions or developmental delays in ROP. Furthermore, hypoxia/ischemia are the key driving force inducing a vascular response, whereby insufficiently perfused tissue is revascularised by the sprouting of new capillaries from pre-existing vessels. However, this revascularization condition is sometimes not successful, leading to the formation of abnormal vessels–so-called ‘neovascularization’. NV is major vision-threatening sequelae in many ischemic retinopathies because of the abnormal vascular leakage, which cause edema and exert tractional forces that untimely cause retinal detachment. It is therefore, tentative to speculate that ischemic vascular disease in the retina may either leave retina permanently ischemic with slow degradation of vision, or alternatively lead to proliferative vascular disease, which can also destroy vision. It has been shown that low concentrations of zinc were neuroprotective in the retina. Is has been demonstrated that the “muffler model” estimated the resting intracellular free Zn^++^ concentration to be 1.07 nM [42], whereas excursions above or below that level may have detrimental effects.

Ischemia is a crucial component of DR [43]. A plethora of reports have addressed ischemic retinopathy and the role in which ZnT8 play in DM. However, the link between ZnT8 and ischemic retinopathy has not been defined yet. Our current investigation also highlights the neuroprotective effects of YC-1. Previously, we have reported the pleiortropic effects of YC-1 on various hypoxia/ischemia-induced retinal pathologies [44], [45], [46]. These investigations have concluded that YC-1 targets several antiangiogenic properties in the ischemic retina, via various mechanisms, which were mainly centered on the; (1) suppression the HIF-1α protein levels, accumulation, and stability; (2) blocking the HIF-1α nuclear shuttling mechanism; (3) promoting the HIF-1α proteasomal degradation; (4) inhibiting hypoxia-inducible factor-2 α (HIF-2α), VEGF, erythropoietin (EPO), enodthelin-1 (ET-1), matrix metalloproteinase-9 (MMP-9), and inducible nitric oxide synthase (iNOS) at the message and the protein levels. Furthermore, our current sequence analysis of ZnT8 indicates the presence of a hypoxia response element (HRE) [46] in the promoter region of ZnT8. Therefore, it is plausible to suggest that HIF-1α (or possibly HIF-2α) overexpression notably suppresses ZnT8 expression, whereas the inhibition of HIF-1α (or possibly HIF-2α) by YC-1 rescues the injured retina by upregulating the ZnT8 levels. It has been demonstrated that levels of platelet-derived growth factor-B (PDGF-B) were increased after ischemic injury [47]. Our studies have previously demonstrated that YC-1 reversed reactive gliosis during ischemic retinal injury via impairing the expression of PDGF-B and glial fibrillary acidic protein (GFAP) in glial cells.

It has been shown that YC-1 had protective effects against sodium nitroprusside-mediated apoptosis in vascular smooth muscle cells [48]. Additionally, it’s been reported that YC-1 protected the white matter axons against damage by ischemia [49]. Studies have indicated that YC-1 was neuroprotective in SH-SY5Y human neuroblastoma cell line against damage caused by MPP+ or 6-OHDA [50]. Moreover, YC-1 was able to inhibit lipopolysaccharide (LPS)-induced iNOS, cyclooxygenase-2 (COX-2) expression, and nuclear factor kappa-b (NFκB) activation, indicating that YC-1 may be developed as an anti-inflammatory neuroprotective agent [50].

Our investigation emphasizes the potential neuroprotective effects of YC-1. It has been demonstrated the antiapoptotic effects of NO are mediated, in part, by cyclic guanosine 3′,5′-cyclic monophosphate (cGMP) and downstream target protein kinase G (PKG) [51]. Furthermore, it has been reported that nitric oxide (NO) serves as neurotransmitter and a neuromodulator in the central and peripheral nervous systems and certain neuronal cells [52]; [53]. At low physiological concentrations, NO can act as an antiapoptotic/prosurvival factor in neural cells [54]. It has been revealed that NO-cGMP-PKG signaling pathway was crucially involved in the learning enhancement of YC-1 and appeared to play an essential role in preventing the activation of a proapoptotic pathway, thus promoting neural cell survival [55]. In addition, in rat astrocytes, YC-1 analogs attenuated H_2_O_2_-induced effects, and demonstrated their neuroprotective effects against cellular injury in cortical neurons [56]. The neuroprotective properties of these analogs may be useful in the treatment of neurodegenerative diseases such as Alzheimer’s disease, Parkinson’s disease and stroke. However, further studies are necessary to determine the exact mechanism by which YC-1 form confers neuroprotection. In the current study, we show for the first time the expression profile of ZnT8 in the retina. Here we show for the first time the expression profile of ZnT8 in the retina. The ONL, OPL, GCL, and NFL tissue layers of the retina exhibited the strongest ZnT8 expression, whereas the PRL, INL and IPL showed moderate ZnT8 immunoreactivity. Furthermore, we demonstrate that hypoxic/ischemic insult has mediated a significant downregulation of ZnT8 at the message and protein levels, *in vitro* and *in vivo*. The ZnT8-depleted cells were primarily localized in the photoreceptor layers/neurosensory retina. Our data also suggest that a treatment with YC-1, a small molecule inhibitor of HIF-1, at a concentration of 100 µM on P12 and P15, rescues the injured retina by restoring the ZnT8 expression to basal homeostatic level, which was comparable to those of the nontreated normoxic retinas. It is noteworthy that although YC-1 inhibition of HIF-1 seems to rescue the ischemia-induced retinal damage, and despite the plethora of published literature available showing the beneficial outcomes of inhibiting HIF-1 [56], [57]; several studies have indicated that inhibition of HIF-1 is detrimental during ischemic or hypoxic injury [58]. These studies have demonstrated that during diabetic complications, the cellular response to hypoxia has been shown to be impaired, and hyperglycemia appears to be the critical event implicated in such deregulation, most likely as a result of destabilization of HIF-1. Hence, impairment of the regulation of HIF-1 may have several deleterious consequences for cell and tissue adaptation and survival at low oxygen levels [58]. This apparent anomaly is difficult to reconcile but possibly related to different experimental models. Taken together, this study extends the possible use of YC-1 as a therapeutic agent in through its effect on Zn^++^ homeostasis. Combined with our *in vivo* finding; our data raises the possibility that under ischemic conditions, glial cells are the predominant source of ZnT8, and YC-1 may be upregulating ZnT8 expression by acting directly on retinal glial cells. Moreover, our data raise an interesting possibility that glial malfunction may play a crucial role in the molecular mechanism(s), which links DM and ischemic retinopathy through the expression of ZnT-8. Whether the reduced expression of ZnT8 is paralleled by significant changes in Zn^++^ homeostasis is yet to be determined. This can be revealed through experiments measuring the intracellular Zn^++^ directly. In addition, ZnT8 knockout animals might prove very useful in addressing this issue. It is however likely, that since the zinc role is intimately linked to AMD, which is an ocular pathology that is manifested during oxygen-induced retinopathy; the possibility exists that reduced ZnT8 expression in our model might be reflected by the significant changes in Zn^++^ homeostasis due to ischemic insults. Moreover, since ZnT8 is responsible for transporting Zn^++^ from the cytosol to the intra-cellular vesicles, it is highly possible that reduced ZnT8 expression may lead to the accumulation of Zn^++^ in the cytosol; hence induce toxicity. This might be supported by the findings of other studies, which demonstrated that in pressure-induced ischemia; zinc has been shown to be toxic and therefore, endogenous zinc may contribute to ischemic neuronal death in the retina [59].

Our data have indicated that treatment with YC-1, a small molecule inhibitor of HIF-1 reversed the decreased ZnT8 expression levels, *in vivo* and *in vitro*. This investigation suggests that YC-1 may play a crucial neuroprotective role against ischemic insult. However, we should highlight that future studies should possibly address the use of different HIF-1 inhibitors in order to solidify our findings.
