# Expression of concern: Zinc transporter 8 (ZnT8) expression is reduced by ischemic insults: A potential therapeutic target to prevent ischemic retinopathy

**DOI:** 10.1371/journal.pone.0227390

**Published:** 2019-12-27

**Authors:** The PLOS ONE Editors

After this article [1] was published, concerns were raised about the quality and presentation of western blot data reported in Figure 4B. Given the brightness/contrast levels of images in this figure and the vertical lines separating lanes, one cannot confirm the integrity of the images or clarify whether the lanes for each experiment show data from the same blot and exposure. Furthermore, the band in lane 7 of the ZnT8 panel appears to be of lower resolution than other bands in the same panel and has abrupt vertical and horizontal edges.

Figure 4C presents densitometric analysis of the western blot, and so the image concerns also have potential implications for the validity of these quantitative data.

The authors were unable to resolve these issues or provide the original data underlying the results in Figure 4B and Figure 4C in response to the journal’s queries. Therefore, the *PLOS ONE* Editors issue this Expression of Concern.

Per FAAM, the underlying data for this study were held by MD for whom current contact information is not available. The journal has not received confirmation that MD received communications about this case.
